# How long were older people expected to live with or without sarcopenia? Multistate modeling of a national cohort study

**DOI:** 10.3389/fpubh.2023.1203203

**Published:** 2023-09-14

**Authors:** Bo Ye, Yujie Wang, Jixiang Xu, Junjia Jiang, Shitong Yang, Jie Chen, Zhijun Bao, Junling Gao

**Affiliations:** ^1^Huadong Hospital, Fudan University, Shanghai, China; ^2^School of Public Health, Fudan University, Shanghai, China; ^3^Collaborative Innovation Cooperative Unit of National Clinical, Shanghai, China; ^4^Core Unit of Shanghai Clinical Research Center for Geriatric, Shanghai, China

**Keywords:** sarcopenia, possible sarcopenia, life expectancy, multistate model, transitions

## Abstract

**Objectives:**

Sarcopenia is well known to be associated with mortality, but there is a lack of evidence on the estimates of life expectancy (LE) for sarcopenia in China. This study aims to estimate total life expectancy (TLE) and sarcopenia-specific LE in community-dwelling older Chinese adults with and without sarcopenia.

**Methods:**

This study included participants aged 60 years and older who enrolled in the cohort in 2011 and 2013 and at least completed one follow-up until 2015 as part of the China Health and Retirement Longitudinal Study (CHARLS). The criteria for defining sarcopenia were based on the guidelines established by the Asian Working Group on Sarcopenia in 2019. TLE and sarcopenia-specific LE were estimated for the total population and subgroups using continuous-time multistate modeling.

**Results:**

A total of 6,029 participants (49.2% women) with an average age of 68.4 (SD: 6.56) years were included in the study. The baseline prevalence of sarcopenia and possible sarcopenia was 19.5 and 44.9%, respectively. We observed that sarcopenia stages naturally deteriorated to worse stages (including death, by 24.4%) and returned to better stages (17.1%) during a median follow-up of 3.92 years (IQR: 2.00 ~ 4.00). The average TLE at the age of 60 was 20.9 [95% CI: 20.2–21.5] years (22.1 [95% CI: 19.6–24.6] for non-sarcopenic older adults, 20.9 [95% CI: 19.5–22.3] for possible sarcopenic, and 18.7 [95% CI: 16.4–21.1] for sarcopenic). Men, former and current smokers, and those living in northwest China had less TLE. Sarcopenic older adults, those with lower education, those who are unmarried, those with agriculture hukou, and those living in rural and northwest China were expected to live fewer years with non-sarcopenia. Sarcopenic older people, men, those with agriculture hukou, and those living in rural and southwest China were expected to live more years with sarcopenia.

**Discussion:**

The results improved our understanding of the relationship between sarcopenia and life expectancy. We suggested that targeted strategies should be considered in high-risk populations and underdeveloped regions to prevent sarcopenia and improve non-sarcopenic life years for the older population.

## Introduction

Sarcopenia is defined as a syndrome characterized by progressive and generalized loss of skeletal muscle mass and strength and/or physical performance, associated with advancing aging (1) and with an increased risk of adverse outcomes such as falls (2), physical disability (3), poor quality of life (4), and mortality (5, 6). It has been formally recognized as a disease with the code M62.84 in the 10th version of the International Classification of Disease (ICD-10) (7). In the community, a growing number of older adults have some degree of physical or cognitive impairment because of the aging population and of increasing life expectancy (LE). The underlying decline of muscle mass or strength is often insufficient for a clinical diagnosis of sarcopenia, yet it hinders functional performance. Compared to people of the same age without sarcopenia, there is also an increased economic burden for individuals, families, and healthcare systems associated with sarcopenia due to the increased risk of hospitalization and higher costs during hospital stays (1).

A recent review concluded that the worldwide prevalence of sarcopenia varies widely depending on different assessment criteria, ranging from 9.9 to 40.4%. The average prevalence was 12.9%, according to the European Working Group on Sarcopenia in Older People/Asian Working Group on Sarcopenia (EWGSOP/AWGS) (8, 9), and it increased with age (10). In China, based on the AWGS criterion, the sarcopenia prevalence among older adults was 14.0%, which was a little higher in Chinese women than in men (11). The total life expectancy (TLE) of the Chinese population has increased to 78.2 years in 2021. It is important to determine whether those years are lived in health or whether the additional years of life result in an expansion of unhealthy status among the older population (12). The standard steps revised by EWGS/AWGS for identifying and diagnosing sarcopenia in older adults presented the results for four sarcopenia states: no sarcopenia, possible sarcopenia, sarcopenia, and severe sarcopenia (13, 14). This provides an important basis for three-grade prevention strategies for sarcopenia in older populations. Considering that sarcopenia is a highly prevalent condition among older adults and that it is associated with multiple adverse outcomes (15), it is necessary to identify whether and when older residents will live with possible sarcopenia.

With the growing awareness of the need for early recognition of musculoskeletal degeneration, primary care physicians and family caregivers have an increasingly important role in the intervention of older adults with possible sarcopenia. Studies showing risk factors related to sarcopenia, such as increasing age, unmarried status, living alone, rural residence, smoking, physical inactivity, malnutrition, and chronic diseases (1, 16–18), can provide health education and specific measures for older audiences. Recent research showed that a difference of over 8 years in TLE was attributable to lifestyle factors for Chinese adults (19). Additionally, easily interpretable ways in terms of LE may be additionally helpful for risk communication with older people and their caregivers. LE for a general older population with or without sarcopenia and stratified by modified risk factors is lacking in China. Moreover, estimates of LE stratified by sex, marital status, education, and other characteristics would be informative for vulnerable population identification and more precise risk communications.

This study aimed to estimate the TLE and sarcopenia-specific LE (NSLE: non-sarcopenic life expectancy; PSLE: possible sarcopenic life expectancy; SLE: sarcopenic life expectancy) among sarcopenic and non-sarcopenic older adults based on a nationwide cohort study from China. We additionally examined LE in older Chinese adults stratified by demographics, lifestyle factors, and regions.

## Methods

### Study design and participants

This study is based on the China Health and Retirement Longitudinal Study (CHARLS), which is a nationally ongoing representative longitudinal survey to examine health and economic adjustments to the rapid aging of the population in China. A more detailed description was published elsewhere (20). Because the CHARLS did not collect physical measures related to sarcopenia assessment for all participants, this study included 6,029 participants aged ≥60 years old who enrolled in the cohort in 2011 and 2013 and at least completed one follow-up until 2015. Given the requirements of the multistate model for sample data, participants were classified into three alive states (no sarcopenia, possible sarcopenia, and sarcopenia) and death in three response waves. Therefore, 6,029, 4,998, and 4,262 participants responded in Wave 1 (survey in 2011–2012), Wave 2 (survey in 2013–2014), and Wave 3 (survey in 2015–2016), respectively. The flowchart of participants is shown in Supplementary Figure S1.

### Assessment of the sarcopenia state

According to the AWGS 2019 algorithm (13), the sarcopenia state was classified into “no sarcopenia,” “possible sarcopenia,” and “sarcopenia,” which was assessed by three components: muscle strength, appendicular skeletal muscle mass (ASM), and physical performance. Sarcopenia was defined as low muscle mass plus low muscle strength or low physical performance. Possible sarcopenia was defined as low muscle strength with or without reduced physical performance. Further details about the definitions for sarcopenia components in the CHARLS have been described elsewhere (16).

#### Muscle strength

Handgrip strength (kg), an indicator of muscle strength, was measured in the dominant hand and the non-dominant hand, with the participant squeezing a YuejianTM WL-1000 dynamometer (Nantong Yuejian Physical Measurement Instrument Co., Ltd., Nantong, China) as hard as possible (20). Every participant was measured twice for both left and right hands by holding the dynamometer at a right angle (90°). The cutoff points for low grip strength were <28 for men and <18 for women, respectively.

#### The ASM

The ASM was estimated by a validated anthropometric equation in Chinese adults (21): ASM = 0.193*body weight + 0.107*height − 4.15*sex − 0.037*age − 2.631. The height, body weight, and age were measured in centimeters, kilograms, and years, respectively. For sex, the value 1 represented men, and the value 2 represented women. The agreement between the ASM equation model and dual X-ray absorptiometry (DXA) was strong (21). After calculating the ASM, the height-adjusted muscle mass (ASM/Ht^2^) was calculated using the ASM divided by the square of the height in meters. According to the AWGS 2019 (13), the cutoff points for defining low muscle mass were <7.0 for men and <5.4 for women.

#### Physical performance

The chair stand test measures the amount of time needed for the participants to rise continuously five times while keeping their arms folded across their chest from a chair. Participants who attempted but failed to perform the chair stand text were considered to have low physical performance for analyses. According to AWGS2019 (13), the criteria for low physical performance is a 5-time chair stand test of ≥12 s.

### Age calculation

The multistate model used to estimate LE needs not only the exact status of a condition but also the exact age in the specific states. The CHARLS recorded the exact birthday of all participants and the exact year and month of interview for all survivors in each wave. The ages of survivors were calculated by the duration of the interview date and their birthdays. For age at death, the exact death time was recorded in Wave 2. Thus, the age of death for those who were dead during Wave 1 and Wave 2 was calculated by the difference between the specific time of death and birthday. In Wave 3, the exact time of death was not recorded. Referring to the previous method (22, 23), the median of the two follow-up times (the specific wave with death information and its former wave) was calculated as the death time, which was used to calculate the age at death.

### Subpopulation identifier

To examine potential factors related to TLE and sarcopenia-specific LE, we classified the whole population into subpopulations by sex (man and woman), education (illiterate, non-formal education, elementary school, and middle school or above), marital status (unmarried and married), and hukou status (agriculture and non-agriculture) by self-reported data in the earliest response. Living residences (rural and urban) and regions (Northeast, East, North, Centre, South, Southwest, and Northwest) were identified according to government office region using *zip* codes in the database. We also examined the effects of behaviors including smoking (no, former, or current) and drinking (yes or no) on TLE and sarcopenia-specific LE.

### Statistical analysis

Descriptive statistics for demographic and lifestyle variables are presented as frequency and percentage, and continuous variables are described as means and standard deviation (SD), and the chi-squared test was used to evaluate the difference between the prevalence of sarcopenia by subgroups. Continuous-time multistate models were used to estimate the TLE and sarcopenia-specific LE by the following two steps. In the first step, a general multistate Markov model by the *msm (multi-state model)* package (24) of the R language (25) was used to examine the parameters of yearly transition probabilities and the state distribution obtained from the general multistate Markov model conditioned on age and identifiers of a subpopulation, as defined in Supplementary Figure S2. In the second step, based on the parameters obtained in the first step, a multistate life table (MSLT) method was used to calculate the TLE and sarcopenia-specific LE by the *elect* (*Estimating Life Expectancies in Continuous Time*) package of R (26). In this study, we assumed that the human maximal age was 120 years old when we ran *elect* with the default “*step*” method for the numerical approximation. For each estimate, a standard error was calculated using a bootstrapping method that executed 30 repeated estimates through random draws. The estimation method of life expectancy was shown in the Supplementary material (p. 4–5).

To assess the robustness of the findings, three methods of sensitivity analysis were used. First, we excluded the sample that was newly enrolled in the second wave because a short follow-up period may affect the robustness of the results. Next, we, respectively, reran elect with the alternative “*MiddleRiemann*” and “*Simpson*” methods for the numerical approximation (26). Windows-based Stata version 14.0 and R version 4.2.1 were used for all of the statistical analysis, and a *p*-value of less than 0.05 was considered to be statistically significant.

## Results

### Study population and prevalence of sarcopenia

The mean age of the analysis sample was 68.4 years (SD = 6.56), with 49.2% being women. The baseline prevalence of sarcopenia and possible sarcopenia were 19.5 and 44.9%, respectively. As shown in Table 1, older people with sarcopenia had a significantly older age (*p* < 0.001). The prevalence of sarcopenia was significantly different in sex (*p* < 0.001), education (*p* < 0.001), marital status (*p* < 0.001), smoking (*p* = 0.006), drinking (*p* < 0.001), hukou type (*p* < 0.001), living residence (*p* < 0.001), and region (*p* < 0.001).

**Table 1:** Prevalence of sarcopenia by participant characteristics at baseline.

Characteristics	Prevalence of sarcopenia, *n* (%)			*p*
	No sarcopenia	Possible sarcopenia	Sarcopenia	
Age, mean (SD)	65.7 (4.73)	68.7 (6.43)	72.7 (7.31)	<0.001
**Sex**				<0.001
Man	1,286 (42.0)	1,297 (42.4)	479 (15.6)	
Woman	860 (29.0)	1,412 (47.6)	695 (23.4)	
**Education**				<0.001
Illiterate	495 (21.9)	1,125 (49.8)	641 (28.4)	
Non-formal education	465 (35.7)	589 (45.2)	249 (19.1)	
Elementary school	643 (43.6)	629 (42.6)	204 (13.8)	
Middle school or above	543 (54.9)	366 (37.0)	80 (8.1)	
**Marital status**				<0.001
Married	286 (21.8)	627 (47.9)	397 (30.3)	
Unmarried	1860 (39.4)	2080 (44.1)	777 (16.5)	
**Smoking**				0.006
No	697 (37.8)	814 (44.1)	333 (18.1)	
Former	286 (37.9)	341 (45.2)	127 (16.8)	
Current	1,159 (34.0)	1,542 (45.2)	711 (20.8)	
**Drinking**				<0.001
No	1,325 (32.0)	1947 (47.0)	873 (21.1)	
Yes	697 (46.0)	591 (39.0)	228 (15.0)	
**Hukou type**				<0.001
Agriculture	1,530 (32.0)	2,217 (46.3)	1,041 (21.7)	
Non-agriculture	591 (49.4)	476 (40.0)	130 (10.9)	
**Living residence**				<0.001
Rural	1,216 (31.0)	1836 (46.8)	867 (22.1)	
Urban	930 (44.1)	873 (41.4)	307 (14.6)	
**Region**				<0.001
Northeast	211 (42.2)	212 (42.4)	77 (15.4)	
East	710 (37.9)	822 (43.8)	343 (18.3)	
North	179 (35.4)	245 (48.4)	82 (16.2)	
Centre	348 (36.6)	422 (44.4)	181 (19.0)	
South	177 (30.8)	264 (45.9)	134 (23.3)	
Southwest	393 (33.2)	534 (45.1)	257 (21.7)	
Northwest	128 (29.2)	210 (48.0)	100 (22.8)	

SD, standard deviation.

### Transitions in sarcopenia stages

**Figure 1:**
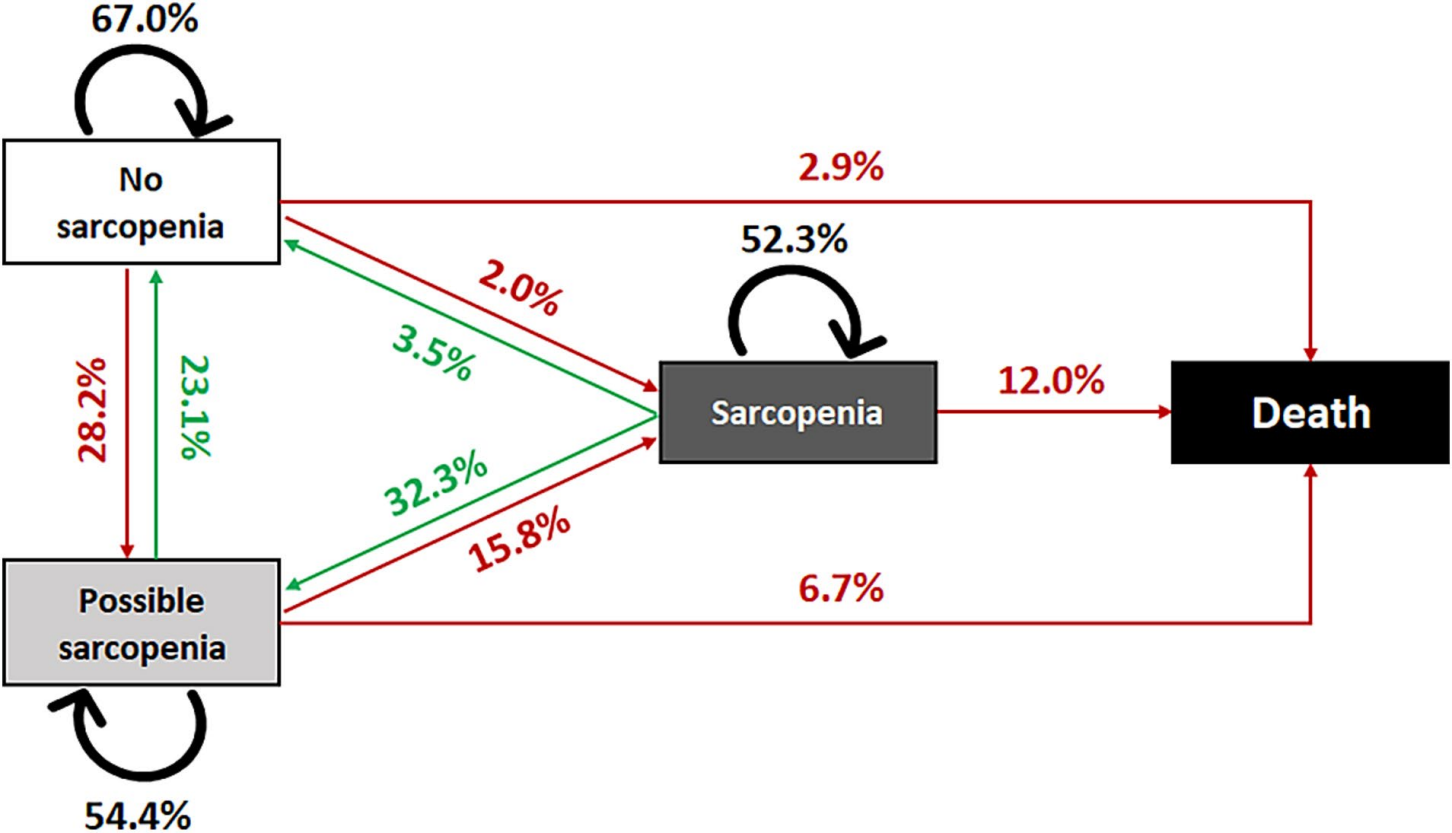
Transitions of sarcopenia state, China Health and Retirement Longitudinal Study, 2011–2015.

As presented in Figure 1, during a median follow-up of 3.92 years (IQR: 2.00 ~ 4.00), 1,119 of 3,386 older people showed transitions from no sarcopenia to possible sarcopenia (28.2%), sarcopenia (2.0%), and death (2.9%); 1,874 of 4,106 from possible sarcopenia to no sarcopenia (23.1%), sarcopenia (15.8%), and death (6.7%); and 844 of 1,768 from sarcopenia to no sarcopenia (3.5%), possible sarcopenia (32.3%), and death (12.0%), respectively. In general, 24.4% of sarcopenic older adults deteriorated to a worse stage, and 17.1% returned to a better one.

### Estimates of LE

**Figure 2:**
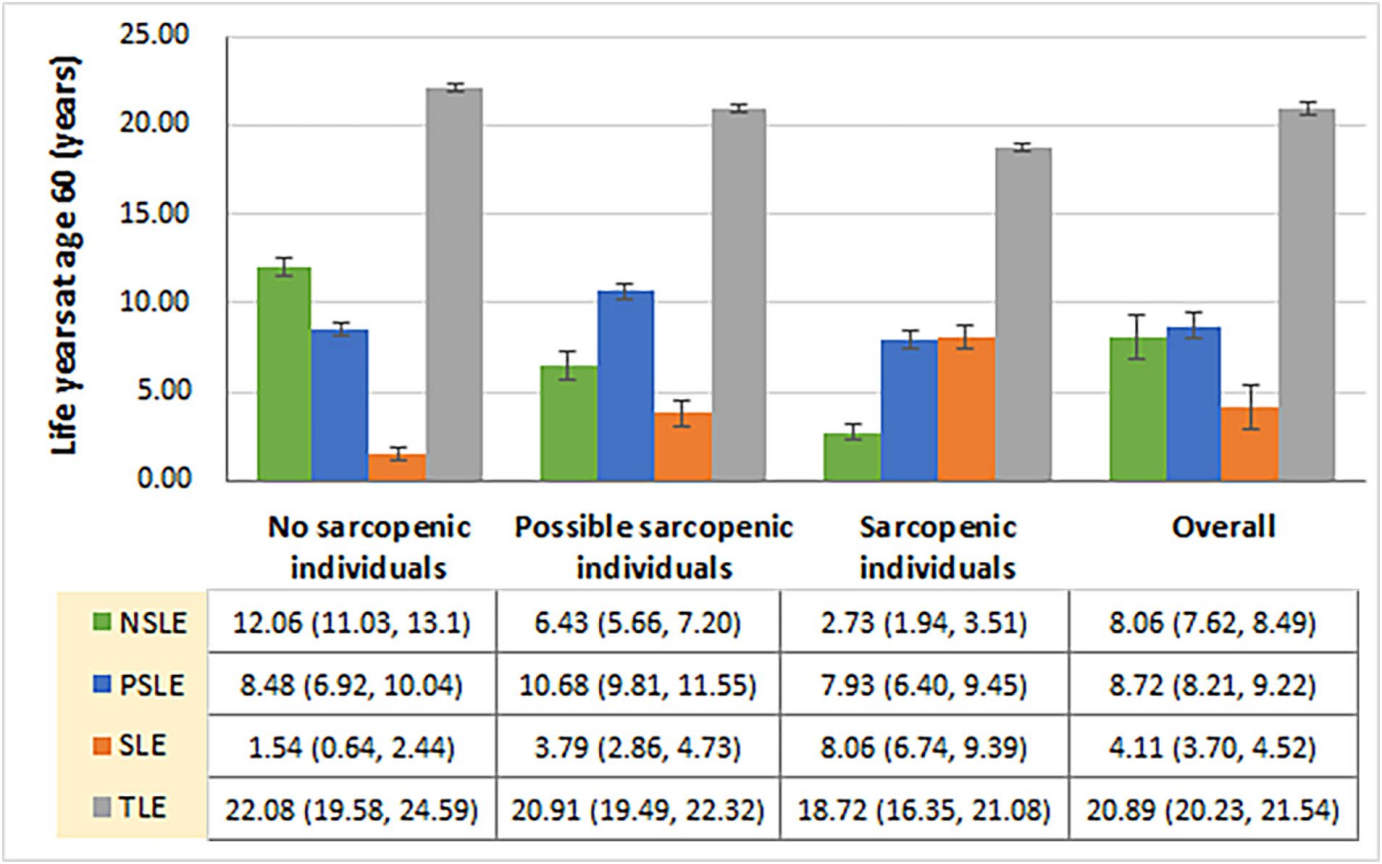
Total and sarcopenia-specific life expectancies among older Chinese with and without sarcopenia at age 60, China Health and Retirement Longitudinal Study, 2011–2015. NSLE, non-sarcopenic life expectancy; PSLE, possible sarcopenic life expectancy; SLE, sarcopenic life expectancy; TLE, total life expectancy.

As shown in Figure 2, TLE at age 60 was, on average, 20.9 years (95% CI: 20.2–21.5) in China overall and 22.1 years (95% CI: 19.6–24.6) for those with no sarcopenia, 20.9 years (95% CI: 19.5–22.3) for possible sarcopenia, and 18.7 years (95% CI: 16.4–21.1) for sarcopenia, respectively. Sarcopenic older adults exhibited a markedly extended sarcopenia life expectancy (SLE) compared to individuals with possible sarcopenia or non-sarcopenia. Specifically, the SLE for sarcopenic older adults (8.1 years [95% CI: 6.7–9.4]) was more than double that of possible sarcopenic individuals (3.8 years [95% CI: 2.9–4.7]) and five times longer than non-sarcopenic individuals (1.5 years [95% CI: 0.6–2.4]). Similarly, sarcopenic older adults had notably shorter non-sarcopenia life expectancy (NSLE) compared to possible sarcopenic individuals and non-sarcopenic individuals. The NSLE for sarcopenic older adults (2.7 years [95% CI: 1.9–3.5]) was approximately half of that for possible sarcopenic individuals (6.4 years [95% CI: 5.7–7.2]) and just a quarter of that for non-sarcopenic individuals (12.1 years [95%CI: 11.0–13.1]).

### Stratified LE

**Figure 3:**
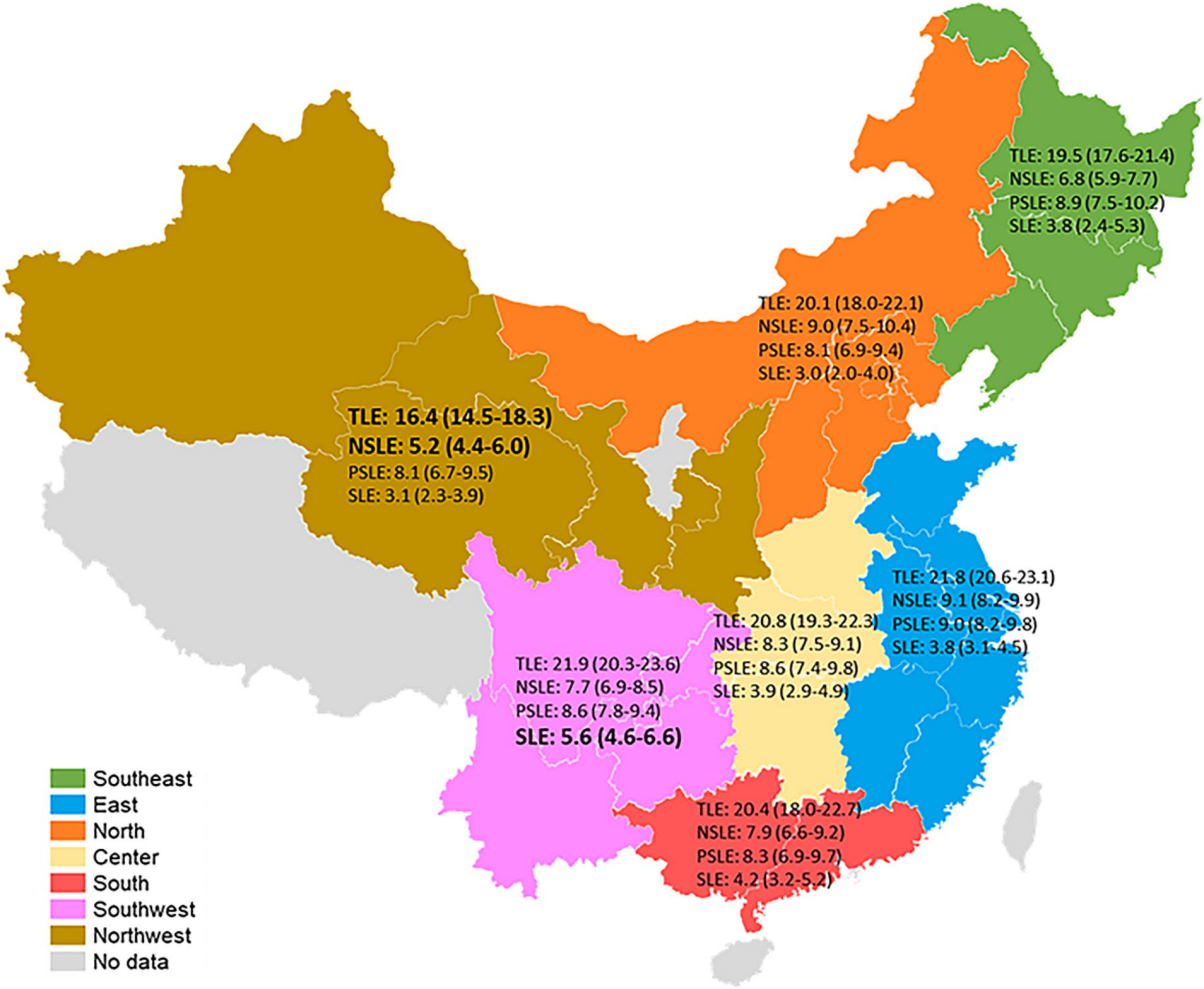
Total and sarcopenia-specific life expectancies among older Chinese at age 60 by regions, China Health and Retirement Longitudinal Study, 2011–2015. TLE, total life expectancy; NSLE, non-sarcopenic life expectancy; PSLE, possible sarcopenic life expectancy; SLE, sarcopenic life expectancy.

Table 2 presents the TLE and sarcopenia-specific LE among demographic, lifestyle, and regional factors. Men (19.3 years [95%CI: 18.4 ~ 20.2]), former smokers (16.7 years [95%CI: 15.3 ~ 18.1]), and current smokers (20.0 years [95%CI: 18.7 ~ 21.2]) were expected to live fewer total life years. Those with lower education (7.1 years [95%CI: 6.5 ~ 7.6] for those with illiterate education and 6.9 years [95%CI: 6.2 ~ 7.6] for those with non-formal education), those who are unmarried (6.8 years [95%CI: 6.1 ~ 7.6]), those with agriculture hukou (7.4 years [95%CI: 7.0 ~ 7.8]), and those living in rural areas (7.4 years [95%CI: 7.0 ~ 7.9]) were expected to live fewer life years with non-sarcopenia. Men (5.5 years [95%CI: 4.8 ~ 6.2]), those with agriculture hukou (4.5 years [95%CI: 4.1 ~ 5.0]), and those living in rural areas (4.8 years [95%CI: 4.3 ~ 5.3]) were expected to live more life years with sarcopenia. In addition, Figure 3 presents the TLE and sarcopenia-specific LE among older Chinese at age 60 by region. Older Chinese living in northwest China (16.4 years [95%CI: 14.5 ~ 18.3]) were expected to live fewer total life years, while those living in the northwest (5.2 years [95%CI: 4.4 ~ 6.0]) of China were expected to live fewer life years with non-sarcopenia, and those living in the southwest of China (5.6 years [95%CI: 4.6 ~ 6.6]) were expected to live more life years with sarcopenia.

**Table 2:** Total and sarcopenia-specific life expectancies for older Chinese at age 60 by demographic and lifestyle subgroups.

Variables	Total and sarcopenia-specific life expectancy, mean (95%CI)			
	NSLE	PSLE	SLE	TLE
**Sex**				
Man	8.38 (7.85, 8.92)	7.87 (7.23, 8.50)	3.05 (2.70, 3.39)	19.30 (18.44, 20.15)
Woman	7.71 (7.15, 8.26)	9.62 (9.04, 10.20)	5.51 (4.82, 6.19)	22.83 (21.80, 23.87)
**Education**				
Illiterate	7.07 (6.53, 7.60)	9.52 (9.00, 10.05)	4.76 (4.18, 5.33)	21.35 (20.55, 22.14)
Non-formal education	6.90 (6.21, 7.60)	8.33 (7.30, 9.35)	4.29 (3.47, 5.10)	19.52 (18.01, 21.02)
Elementary school	9.04 (8.10, 9.98)	8.21 (7.24, 9.19)	3.41 (2.47, 4.36)	20.67 (19.11, 22.23)
Middle school or above	10.03 (9.18, 10.88)	8.88 (7.59, 10.16)	3.22 (2.14, 4.30)	22.12 (19.96, 24.28)
**Marital status**				
Married	8.41 (7.91, 8.90)	8.67 (8.09, 9.24)	3.96 (3.55, 4.37)	21.03 (20.16, 21.90)
Unmarried	6.85 (6.14, 7.56)	8.95 (8.09, 9.80)	4.47 (3.79, 5.15)	20.26 (19.09, 21.44)
**Smoking**				
No	8.36 (7.88, 8.83)	9.70 (9.05, 10.35)	5.02 (4.43, 5.61)	23.08 (21.99, 24.16)
Former	8.09 (7.02, 9.16)	6.71 (5.64, 7.77)	1.90 (1.31, 2.48)	16.69 (15.26, 18.12)
Current	7.61 (6.85, 8.36)	8.33 (7.59, 9.08)	4.03 (3.34, 4.71)	19.97 (18.74, 21.20)
**Drinking**				
No	7.82 (7.35, 8.29)	8.99 (8.55, 9.43)	4.30 (3.87, 4.74)	21.11 (20.37, 21.86)
Yes	8.62 (7.84, 9.41)	7.98 (7.00, 8.96)	3.75 (3.07, 4.44)	20.36 (18.90, 21.82)
**Hukou type**				
Agriculture	7.36 (6.95, 7.77)	8.86 (8.46, 9.27)	4.52 (4.09, 4.95)	20.74 (20.04, 21.44)
Non-agriculture	10.61 (9.61, 11.61)	7.94 (6.74, 9.15)	2.57 (1.96, 3.18)	21.12 (19.51, 22.74)
**Living residence**				
Rural	7.43 (6.98, 7.88)	8.80 (8.36, 9.25)	4.83 (4.33, 5.33)	21.07 (20.28, 21.86)
Urban	9.33 (8.59, 10.06)	8.39 (7.49, 9.29)	2.83 (2.39, 3.27)	20.55 (19.36, 21.74)

CI, confidence interval; NSLE, non-sarcopenic life expectancy; PSLE, possible sarcopenic life expectancy; SLE, sarcopenic life expectancy; TLE, total life expectancy.

### Sensitive analysis

The results of sensitive analyses conducted by different methods were almost consistent with the main analysis (Supplementary Tables S2–S7). There were almost no differences from the results of the main analyses, which indicated the robustness of the findings.

## Discussion

The findings of our study suggest that sarcopenia stages can transition into each other naturally. Sarcopenia stages can deteriorate to a worse stage (including death, 24.4%) but can also turn back to a better stage (17.1%). The majority stayed in the same stages, which is consistent with the previous study, even with a longer follow-up (27) and more stages (28). These results provide evidence that sarcopenia is a dynamic, reversible, and generally progressive disease similar to frailty (29, 30). Beyond the age of 50, muscle mass and strength started to decline smoothly with age and showed a steep drop over 85 years (1, 14). Possible sarcopenia is a relatively unstable stage, in that approximately half of the patients transition to other stages, indicating a possibility for intervention. For example, nutrition and exercise would provide benefits for sarcopenic patients (13, 31). Therefore, it is probably the healthy lifestyle approach advocated by health authorities in recent years that promotes the recovery of some sarcopenic patients to a certain extent. It is well known that sarcopenia is correlated with a higher risk of mortality (1, 5). We also found that older adults with possible sarcopenia or sarcopenia have an approximately 2.9-fold higher risk of all-cause mortality than those without, which is similar to the newly available evidence from a meta-analysis based on community-dwelling older adults (5).

Our study shows significant differences in TLE and sarcopenia-specific LE between sarcopenic and non-sarcopenic older people. In both absolute and relative terms, at age 60 years, sarcopenic older Chinese expected to live more life years with sarcopenia (8.06 vs. 1.54 years; 43.0% vs. 7.0%), fewer life years with non-sarcopenia (2.73 vs. 12.06 years; 14.6% vs. 54.6%), and fewer total life years (18.72 vs. 22.08 years), compared to non-sarcopenic individuals. Compared to the average level, the findings of a 10.4% reduction in TLE, a double increase in SLE, and an almost two-thirds decrease in NSLE for sarcopenic older adults are amazing but understandable. Sarcopenia has been proven to lead to multiple adverse health outcomes, including falls, frailty, functional decline, poor quality of life, and mortality (1). It is not difficult to understand that these adverse outcomes, in turn, can reduce older patients’ physical activity and/or social participation, creating an adverse cycle. Therefore, we suggest that sarcopenic older adults need to be intervened with and treated as soon as possible to reduce the impact on their daily activities.

The life expectancy results for older Chinese at age 60 by population subgroups suggest that women were expected to live more total life years but also more life years with sarcopenia and possible sarcopenia than men. This paradoxical phenomenon was also found in many previous studies that showed that women lived longer but were unhealthy (1, 11, 19). Both former and current smokers were expected to live fewer total years, which is consistent with previous studies (31). In the current study, those who start smoking in early adult life and continue smoking lose approximately 3.11–6.39 years of life. A study conducted in Japan showed that the reduced life years of those smoking people were almost a decade (32). We also found that those with lower education and those who are unmarried were expected to live fewer life years with non-sarcopenia. In common with previous studies, lower education and unmarried status were risk factors for sarcopenia (18, 33).

Poorer population health tends to be present in poorer regions (34). The results of our study also show that older people with agriculture hukou and those living in rural and northwest China were expected to live fewer life years with non-sarcopenia and those with agriculture hukou, those living in rural and southwest China were expected to live more life years with sarcopenia. In China, the development of the regional economy is unbalanced. The eastern region has the fastest economic development, while the economies of the western regions are relatively backward. It may be explained by the fact that health inequality is closely related to regional economic inequality (34). Hukou type presents an individual’s birthplace; that is, agriculture hukou presents the person born in a rural region. Although some people with agriculture hukou live in urban areas, childhood experiences may influence physical function in later life (35). Previous studies have shown that the prevalence of sarcopenia is higher in rural regions (16, 36). This may be due to inadequate medical conditions and a higher incidence of malnutrition in the rural population (36).

The strengths of this study include the national sample and the measurement of sarcopenia according to the AWGS 2019, which enhance the representativeness of the results and their comparability to other studies. TLE and sarcopenia-specific LE were estimated using the multistate model, which can also describe how individuals move through different stages of sarcopenia and can capture the dynamic nature of sarcopenia, compared with the existing methods of calculating LE, such as the Cox model and the Sullivan method. To our knowledge, this study is one of the few to profile the transitions of sarcopenia stages and their end to death and the first to present the relationship between expected life years and sarcopenia in older Chinese adults.

However, several limitations are associated with the measurement of sarcopenia in the current study. First, although this is a large national cohort study, the excluded participants due to missing sarcopenia assessments and the loss of follow-up in the study may lead to potential selection bias. More number of older participants in the excluded sample may lead to an underestimation of the prevalence of sarcopenia because of the positive association between sarcopenia and age (10). Second, we estimated the ASM based on an anthropometric equation instead of the DXA, which was recommended by the AWGS 2019 (13). The equation has been validated and shows high agreement with the measurement of DXA in the Chinese population (21).

## Conclusion

The findings of this study present the nature of the transitions of sarcopenia and the estimates of life expectancy with and without sarcopenia. The results improve the understanding of the relationship between sarcopenia and life expectancy, provide a simple measure of health education for older adults, and highlight the importance of early identification and intervention for sarcopenia among older Chinese adults. We suggest that women, older adults with lower education, and those who are unmarried, those with agriculture hukou, and both former and current smokers are particularly susceptible to sarcopenia. Targeted strategies should be taken in rural areas and underdeveloped regions of western China to prevent sarcopenia and improve non-sarcopenic life years for the older population.

## Data availability statement

Publicly available datasets were analyzed in this study. This data can be found here: http://charls.pku.edu.cn/en/.

## Ethics statement

The studies involving human participants were reviewed and approved by Biomedical Ethics Review Committee of Peking University (IRB00001052-11015). The Ethics Committee waived the requirement of written informed consent for participation. This study is a secondary analysis of the publicly available database of CHARLS, and the data have been fully de-identified.

## Author contributions

BY analyzed and interpreted the data, drafted and revised the manuscript. JG designed the study, interpreted the data, and revised the manuscript. YW, JX, JJ, and SY contributed to the visualization and revised the manuscript. ZB and JC contributed to interpreting the data. All authors contributed to the article and approved the submitted version.

## Funding

This study was supported by the National Key Research and Development Program of China (grant: 2018YFC2002000; 2018YFC2002001; 2018YFC2002002), the National Natural Science Foundation of China (grant: 82173634; 82204166), and the Shanghai Clinical Research Center for Aging and Medicine (grant: 19MC1910500).

## Conflict of interest

The authors declare that the research was conducted in the absence of any commercial or financial relationships that could be construed as a potential conflict of interest.

## Publisher’s note

All claims expressed in this article are solely those of the authors and do not necessarily represent those of their affiliated organizations, or those of the publisher, the editors and the reviewers. Any product that may be evaluated in this article, or claim that may be made by its manufacturer, is not guaranteed or endorsed by the publisher.
