# Supplementary material for: How long were older people expected to live with or without sarcopenia? Multistate modeling of a national cohort study
Source: Front Public Health. 2023 Sep 14;11:1203203. doi: 10.3389/fpubh.2023.1203203 (PMC10539905; doi:10.3389/fpubh.2023.1203203)
Supplement: Supplementary file 1 [file Data_Sheet_1.pdf]

Supplementary materials

CONTENT

1. Participants in this study 2

Figure S1. Flowchart of participants 2

Table S1. Comparison of analysis and non-analysis sample at baseline 3

2. Estimation methods of life expectancy 4

Figure S2. Multistate model of sarcopenia state life expectancy 4

3. Sensitive analysis 6

Table S2. Total and sarcopenia-state life expectancy by sarcopenia state excluding newly participants from wave 2 for baseline 8

Table S3. Total and sarcopenia-specific life expectancy for older Chinese at age 60 by demographic and lifestyle subgroups excluding newly participants from wave 2 for baseline. 8

Table S4. Total and sarcopenia-state life expectancy by sarcopenia state using *MiddleRiemann* method 9

Table S5. Total and sarcopenia-specific life expectancy for older Chinese at age 60 by demographic and lifestyle subgroups using *MiddleRiemann* method........ 9

Table S6. Total and sarcopenia-state life expectancy by sarcopenia state using *Simpson* method........ 10

Table S7. Total and sarcopenia-specific life expectancy for older Chinese at age 60 by demographic and lifestyle subgroups using *Simpson* method........ 10

1. Participants in this study

This study is based on the China Health and Retirement Longitudinal Study (CHARLS), which is a nationally ongoing representative longitudinal study of individuals over age 45 in China. CHARLS is supported by Peking University, the National Natural Science Foundation of China, the Behavioral and Social Research Division of the National Institute on Aging, and the World Bank. CHARLS shares the same basic guidelines as the Health and Retirement Study (HRS) and related aging surveys, such as the English Longitudinal Study of Aging (ELSA) in England) and the Survey of Health, Aging and Retirement in Europe (SHARE) in Europe and Israel.

The first wave of CHARLS was conducted between June 2011 and March 2012. The sample population was selected as part of a stratified, multistage probability design. This initial sample included 17708 respondents in 10257 households in 450 villages/urban communities in 150 counties/districts in 28 provinces. The second wave was conducted between July 2013 and January 2014 with following up 15185 participants from the first wave, and newly enrolled 3426 individuals aged 45 and above. The third wave was conducted between July 2015 and January 2016. Given the missing data of sarcopenia assessment and the loss of follow-up, this study included participants aged ≥60 years old who enrolled the cohort in 2011 (wave 1) and 2013 (wave 2), and at least completed one follow-up until 2015 (wave 3). The flowchart of participants is below (**Figure S1**).

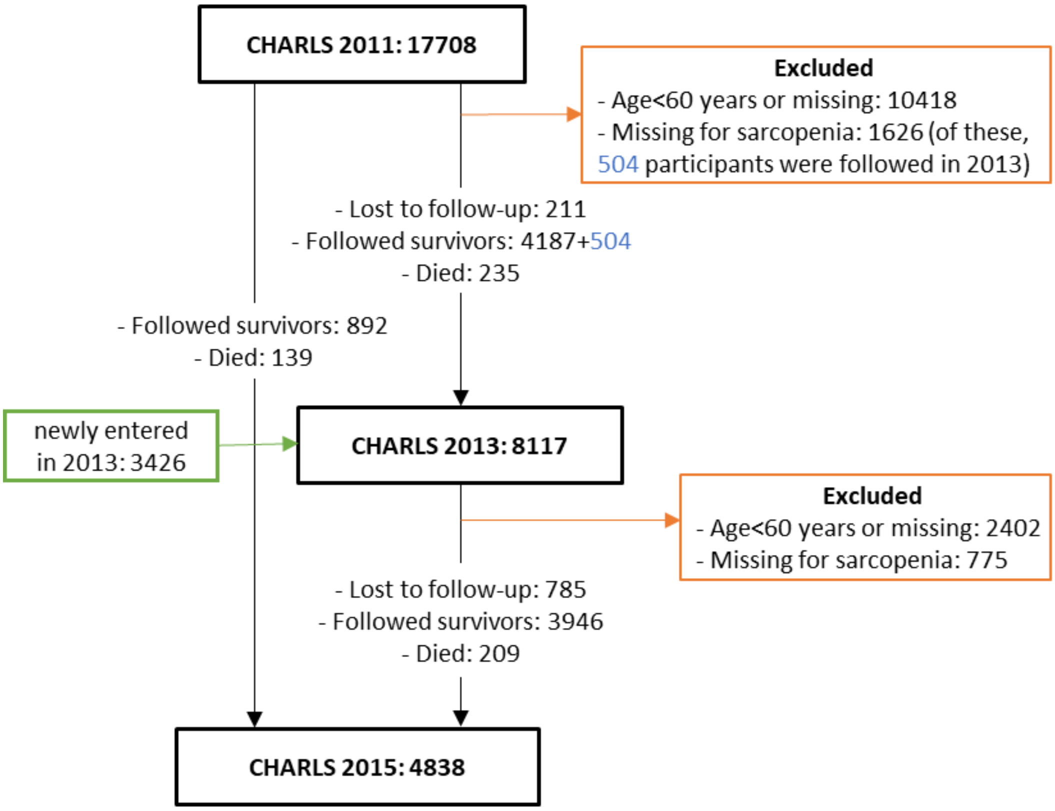

Figure S1. Flowchart of participants

The baseline sample for fitting the multistate model was consist of 5609 participants surveyed in the

first wave and 420 participants newly surveyed in the second wave. To be specific, 1) 3231 participants who entered in 2011 were both followed up in 2013 and 2015 (include 161 dead); 2) 843 participants who entered in 2011 were followed up in 2013 but not in 2015 (include 235 dead); 3) 1031 participants who entered in 2011 were followed up in 2015 but lost to follow-up or missing for sarcopenia assessment in 2013 (include 139 dead); 4) 504 participants who entered in 2011 were both followed up in 2013 and 2015, but were missing for sarcopenia assessment in 2011 (include 32 dead); 5) 420 participants who entered in 2013 were followed up in 2015 (include 16 dead). The characteristics of the analysis sample and the comparison of analysis and non-analysis sample at baseline was showed in **Table S1**.

Table S1. Comparison of analysis and non-analysis sample at baseline

Characteristics	Sample at baseline, n (%)		*P*
	**analysis sample (n=6029)**	**non-analysis sample (n=2285)**	
**Age**, mean (SD)	68.4 (6.56)	69.9 (7.73)	<0.001
**Age group, years**			<0.001
60-65	2352 (39.0)	827 (36.2)	
65-69	1567 (26.0)	474 (20.7)	
70-74	1058 (17.5)	395 (17.3)	
75-79	675 (11.2)	320 (14.0)	
≥80	377 (6.3)	269 (11.8)	
**Sex**			0.487
Man	3062 (50.8)	1141 (49.9)	
Woman	2967 (49.2)	1144 (50.1)	
**Education**			<0.001
Illiterate	2261 (37.5)	780 (34.2)	
Nonformal education	1303 (21.6)	365 (16.0)	
Elementary school	1476 (24.5)	461 (20.2)	
Middle school or above	989 (16.4)	675 (29.6)	
**Marital status**			0.131
Married	1310 (21.7)	531 (23.3)	
Unmarried	4717 (78.3)	1750 (76.7)	
**Smoking**			<0.001
No	1844 (30.7)	484 (21.8)	
Former	754 (12.6)	374 (16.9)	
Current	3412 (56.8)	1358 (61.3)	
**Drinking**			0.009
No	4145 (73.2)	1593 (76.2)	
Yes	1516 (26.8)	499 (23.8)	
***Hukou* type**			<0.001
Agriculture	4788 (80.0)	1291 (57.6)	
Non-agriculture	1197 (20.0)	951 (42.4)	
**Living residence**			<0.001
Rural	3919 (65.0)	875 (38.3)	
Urban	2110 (35.0)	1410 (61.7)	
**Region**			<0.001
Northeast	500 (8.3)	293 (12.8)	
East	1875 (31.1)	695 (30.4)	
North	506 (8.4)	248 (10.8)	
Centre	951 (15.8)	283 (12.4)	
South	575 (9.5)	214 (9.4)	
Southwest	1184 (19.6)	407 (17.8)	
Northwest	438 (7.3)	145 (6.4)	

2. Estimation methods of life expectancy

In this study, sarcopenia-specific life expectancy was estimated based on a time-continuous multi-state model by *elect*[1] and *msm*[2] package of R[3]. This method allows users to compute life expectancies for any number of states and is not limited to progressive processes.

A multi-state model including 3 alive state (no sarcopenia: state 1, possible sarcopenia: state 2, sarcopenia: state 3) and a single absorbing state (death: state 4) was defined as shown in **Figure S2**. The model permits transitions (shown with arrows) from any alive state to any state (including the same state and the dead state).

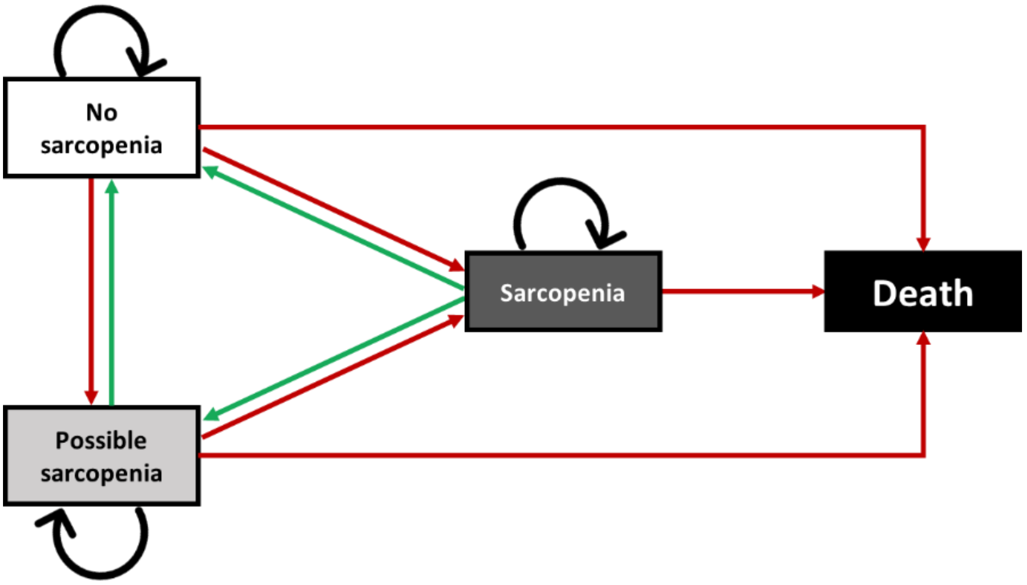

Figure S2. Multistate model of sarcopenia state life expectancy

Let the finite state space be given by {1,2,…,D} where D is the dead state. Let Y_t denote the state at age t and let x denote the time-independent vector with covariate values.

Life expectancy in living state s given state r at age t, for $r, s \in \{1,2,\dots,D-1\}$, is defined by

$$e_{rs}(t|\boldsymbol{x}) = \int_0^{\infty} \mathbb{P}\left(Y_{t+u} = s|Y_t = r, \boldsymbol{x}\right)du \qquad (1)$$

Where $\mathbb{P}(Y_{t+u} = s|Y_t = r, \boldsymbol{x})$ is the transition probability pf being in state s at age $t+u$, given starting state r at age t and covariate values $\boldsymbol{x}$. Marginal life expectancy in state s is irrespective of the initial state at age t and is defined by

$$e_{\cdot s}(t|\boldsymbol{x}) = \sum_{r\neq 4} \mathbb{P}\left(Y_t = r|\boldsymbol{x}\right)e_{rs}(t|\boldsymbol{x}) \qquad (2)$$

Where $\mathbb{P}(Y_t = r|\boldsymbol{x})$ is the probability of being in state r at age t for r$\in$ 1,2, … , D − 1. Total life expectancy at age t is defined as

$$e(t|\boldsymbol{x}) = \sum_{s\neq 4} e_{\cdot s}(t|\boldsymbol{x}) \qquad (3)$$

To be able to estimate life expectancy, transition probabilities and the state distribution are estimated using longitudinal data. Using the same notation as above, we assume that data for individual i and observation j are given by (y_{ij}, t_{ij}, x_i), for $i \in \{1,\ldots,N\}$ and $j \in \{1,\ldots,n_i\}$. Transition probabilities are derived from a multi-state model where the hazards are defined by

$$h_{rs}(t_{ij}) = exp(\beta_{rs} + \xi_{rs}t_{ij} + \gamma_{rs}x_i) \qquad (4)$$

This model is estimated using *msm* package.

The distribution of the state at age t is modelled using a multinomial regression model defined by

$$\mathbb{P}(Y_t = r|\boldsymbol{x}) = \frac{exp(\eta_r(t))}{1+\sum_{r\neq D} exp(\eta_r(t))} \qquad (5)$$

with $\eta_r(t) = \alpha_{r0} + \alpha_{r1}t + \alpha_{r2}x$

for r$\in\{1, 2, ..., D-1\}$. By restricting $\alpha_{10} = \alpha_{11} = \alpha_{12} = 0$, we make r=1 the reference category. This model is estimated in *elect* using the function *multinom* in the package *nnet.*

Life expectancies (1), (2), and (3) can be derived by *elect* package using the parameters in the multi-state model and the multinomial regression model.

1. van den Hout A, Sum Chan M, Matthews F. Estimation of life expectancies using continuous-time multi-state models. Comput Methods Programs Biomed 2019; 178: 11-8.
2. Jackson C. Multi-State Models for Panel Data: The msm Package for R. Journal of Statistical Software 2011; 38(8): 1-28.
3. Team RC. R: A language and environment for statistical computing. 2022. https://www.R-project.org/.

Table S2. Total number of observed transitions in participants categorized by sarcopenia stages over a 4-year follow-up.

	To			
	**No sarcopenia**	**Possible sarcopenia**	**Sarcopenia**	**Death**
**From**				
**No sarcopenia**	2267	954	68	97
**Possible sarcopenia**	950	2232	650	274
**Sarcopenia**	61	571	924	212

Note: Individuals could have experienced one or more than one transition.

3. Sensitive analysis

To assess the robustness of the findings, three methods of sensitivity analysis were used. First, we excluded the sample that newly enrolled in the second wave. The baseline sample for fitting the multistate model was 5609 participants surveyed in the first wave and at least completed one follow-up until 2015. Next, we respectively reran elect with the alternative "*MiddleRiemann*" and "*Simpson*" methods for the numerical approximation, respectively. The results were presented in **Table S3-S8**.

Table S3. Total and sarcopenia-state life expectancy by sarcopenia state excluding newly participants from wave 2 for baseline

Sarcopenia state	Total and sarcopenia-state life expectancy, years [mean (95%CI)]			
	NSLE	PSLE	SLE	TLE
**No sarcopenia**	11.96 (10.93, 12.99)	8.43 (6.87, 9.99)	1.51 (0.63, 2.39)	21.90 (19.42, 24.38)
**Possible sarcopenia**	6.35 (5.59, 7.12)	10.64 (9.77, 11.51)	3.77 (2.85, 4.70)	20.77 (19.37, 22.17)
**Sarcopenia**	2.72 (1.94, 3.51)	7.94 (6.42, 9.47)	8.09 (6.76, 9.42)	18.76 (16.39, 21.12)
**Average**	7.95 (7.51, 8.39)	8.66 (8.16, 9.17)	4.11 (3.70, 4.52)	20.73 (20.07, 21.38)

CI: confidence interval; TLE: Total life expectancy; NSLE: Non-sarcopenic life expectancy; PSLE: Possible sarcopenic life expectancy; SLE: Sarcopenic life expectancy.

Table S4. Total and sarcopenia-specific life expectancy for older Chinese at age 60 by demographic and lifestyle subgroups excluding newly participants from wave 2 for baseline

Variables	Total and sarcopenia-specific life expectancy, years [mean (95%CI)]			
	NSLE	PSLE	SLE	TLE
**Sex**				
Man	8.22 (7.68, 8.75)	7.87 (7.22, 8.52)	3.07 (2.72, 3.42)	19.16 (18.29, 20.02)
Woman	7.69 (7.12, 8.25)	9.50 (8.91, 10.09)	5.46 (4.78, 6.14)	22.64 (21.61, 23.67)
**Education**				
Illiterate	7.01 (6.47, 7.55)	9.41 (8.87, 9.94)	4.75 (4.17, 5.33)	21.17 (20.36, 21.97)
Nonformal education	6.78 (6.08, 7.48)	8.35 (7.30, 9.40)	4.30 (3.47, 5.13)	19.43 (17.90, 20.96)
Elementary school	9.00 (8.05, 9.95)	8.23 (7.23, 9.22)	3.45 (2.48, 4.42)	20.68 (19.08, 22.27)
Middle school or above	9.75 (8.91, 10.59)	8.75 (7.48, 10.02)	3.20 (2.13, 4.26)	21.69 (19.57, 23.81)
**Marital status**				
Married	8.28 (7.78, 8.78)	8.61 (8.03, 9.19)	3.95 (3.54, 4.36)	20.85 (19.98, 21.72)
Unmarried	6.79 (6.07, 7.51)	8.88 (8.01, 9.75)	4.46 (3.77, 5.15)	20.13 (18.95, 21.31)
**Smoking**				
No	8.31 (7.83, 8.80)	9.56 (8.91, 10.21)	5.02 (4.42, 5.61)	22.89 (21.81, 23.97)
Former	7.84 (6.78, 8.90)	6.83 (5.74, 7.92)	1.92 (1.32, 2.52)	16.59 (15.14, 18.03)
Current	7.48 (6.73, 8.23)	8.33 (7.57, 9.08)	3.99 (3.31, 4.68)	19.80 (18.57, 21.03)
**Drinking**				
No	7.74 (7.26, 8.22)	8.90 (8.46, 9.35)	4.30 (3.86, 4.74)	20.95 (20.20, 21.69)
Yes	8.41 (7.63, 9.20)	8.04 (7.03, 9.04)	3.76 (3.06, 4.45)	20.21 (18.73, 21.69)
***Hukou* type**				
Agriculture	7.29 (6.87, 7.71)	8.85 (8.44, 9.26)	4.53 (4.10, 4.97)	20.67 (19.97, 21.38)
Non-agriculture	10.45 (9.44, 11.45)	7.69 (6.50, 8.88)	2.56 (1.94, 3.17)	20.69 (19.10, 22.29)
**Living residence**				
Rural	7.36 (6.90, 7.82)	8.79 (8.34, 9.24)	4.82 (4.31, 5.32)	20.96 (20.17, 21.76)
Urban	9.17 (8.44, 9.91)	8.25 (7.34, 9.16)	2.86 (2.41, 3.31)	20.28 (19.09, 21.48)
**Region**				
Northeast	6.79 (5.89, 7.68)	8.67 (7.36, 9.99)	3.81 (2.36, 5.26)	19.27 (17.42, 21.12)
East	8.92 (8.08, 9.75)	8.93 (8.15, 9.72)	3.94 (3.18, 4.70)	21.79 (20.53, 23.06)
North	8.98 (7.48, 10.47)	8.01 (6.78, 9.25)	2.95 (1.93, 3.98)	19.94 (17.89, 21.99)
Centre	8.15 (7.34, 8.95)	8.61 (7.43, 9.79)	3.95 (2.97, 4.92)	20.70 (19.21, 22.19)
South	7.81 (6.50, 9.12)	8.28 (6.86, 9.71)	4.05 (3.11, 4.99)	20.15 (17.76, 22.53)
Southwest	7.66 (6.83, 8.49)	8.56 (7.71, 9.40)	5.51 (4.50, 6.53)	21.73 (20.06, 23.39)
Northwest	5.00 (4.26, 5.74)	8.10 (6.74, 9.46)	3.08 (2.30, 3.87)	16.18 (14.29, 18.07)

CI: confidence interval; TLE: Total life expectancy; NSLE: Non-sarcopenic life expectancy; PSLE: Possible sarcopenic life expectancy; SLE: Sarcopenic life expectancy.

Table S5. Total and sarcopenia-state life expectancy by sarcopenia state using *MiddleRiemann* method

Sarcopenia state	Total and sarcopenia-state life expectancy, years [mean (95%CI)]			
	**NSLE**	**PSLE**	**SLE**	**TLE**
**No sarcopenia**	12.03 (10.99, 13.06)	8.48 (6.91, 10.04)	1.54 (0.65, 2.44)	22.04 (19.54, 24.55)
**Possible sarcopenia**	6.41 (5.64, 7.17)	10.67 (9.80, 11.54)	3.79 (2.85, 4.73)	20.86 (19.45, 22.28)
**Sarcopenia**	2.72 (1.93, 3.50)	7.91 (6.39, 9.43)	8.06 (6.74, 9.38)	18.68 (16.32, 21.04)
**Average**	8.03 (7.59, 8.46)	8.71 (8.20, 9.21)	4.11 (3.70, 4.52)	20.85 (20.19, 21.50)

CI: confidence interval; TLE: Total life expectancy; NSLE: Non-sarcopenic life expectancy; PSLE: Possible sarcopenic life expectancy; SLE: Sarcopenic life expectancy.

Table S6. Total and sarcopenia-specific life expectancy for older Chinese at age 60 by demographic and lifestyle subgroups using *MiddleRiemann* method

Variables	Total and sarcopenia-specific life expectancy, years [mean (95%CI)]			
	**NSLE**	**PSLE**	**SLE**	**TLE**
**Sex**				
Man	8.35 (7.82, 8.89)	7.85 (7.22, 8.49)	3.05 (2.70, 3.39)	19.26 (18.40, 20.12)
Woman	7.68 (7.12, 8.24)	9.60 (9.02, 10.18)	5.51 (4.82, 6.19)	22.79 (21.76, 23.82)
**Education**				
Illiterate	7.04 (6.51, 7.57)	9.51 (8.98, 10.03)	4.76 (4.18, 5.33)	21.31 (20.51, 22.10)
Nonformal education	6.88 (6.18, 7.57)	8.31 (7.29, 9.34)	4.29 (3.47, 5.10)	19.48 (17.98, 20.98)
Elementary school	9.01 (8.08, 9.95)	8.20 (7.23, 9.18)	3.41 (2.47, 4.36)	20.63 (19.07, 22.19)
Middle school or above	10.00 (9.15, 10.84)	8.87 (7.58, 10.15)	3.22 (2.14, 4.3)	22.08 (19.92, 24.24)
**Marital status**				
Married	8.38 (7.88, 8.88)	8.65 (8.07, 9.23)	3.96 (3.55, 4.37)	20.99 (20.11, 21.86)
Unmarried	6.83 (6.12, 7.53)	8.93 (8.08, 9.78)	4.47 (3.79, 5.15)	20.23 (19.05, 21.4)
**Smoking**				
No	8.33 (7.85, 8.80)	9.69 (9.04, 10.34)	5.02 (4.43, 5.61)	23.03 (21.95, 24.12)
Former	8.06 (6.99, 9.13)	6.70 (5.64, 7.76)	1.90 (1.31, 2.48)	16.66 (15.23, 18.08)
Current	7.58 (6.83, 8.34)	8.32 (7.58, 9.06)	4.03 (3.34, 4.71)	19.93 (18.70, 21.16)
**Drinking**				
No	7.79 (7.32, 8.26)	8.98 (8.53, 9.42)	4.30 (3.86, 4.74)	21.07 (20.33, 21.82)
Yes	8.59 (7.81, 9.38)	7.97 (6.99, 8.95)	3.75 (3.07, 4.44)	20.32 (18.86, 21.78)
***Hukou* type**				
Agriculture	7.34 (6.92, 7.75)	8.85 (8.45, 9.25)	4.52 (4.09, 4.95)	20.70 (20.00, 21.40)
Non-agriculture	10.58 (9.58, 11.58)	7.94 (6.73, 9.14)	2.57 (1.96, 3.18)	21.08 (19.47, 22.70)
**Living residence**				
Rural	7.41 (6.96, 7.86)	8.79 (8.35, 9.23)	4.83 (4.33, 5.33)	21.03 (20.24, 21.81)
Urban	9.30 (8.56, 10.03)	8.38 (7.48, 9.28)	2.83 (2.39, 3.27)	20.51 (19.32, 21.70)
**Region**				
Northeast	6.78 (5.91, 7.66)	8.85 (7.52, 10.18)	3.83 (2.36, 5.29)	19.46 (17.59, 21.33)
East	9.03 (8.20, 9.86)	8.98 (8.20, 9.76)	3.79 (3.08, 4.51)	21.80 (20.57, 23.04)
North	8.93 (7.45, 10.40)	8.10 (6.86, 9.34)	3.00 (1.97, 4.03)	20.03 (17.99, 22.07)
Centre	8.29 (7.48, 9.10)	8.60 (7.42, 9.78)	3.91 (2.93, 4.89)	20.80 (19.30, 22.30)
South	7.85 (6.58, 9.12)	8.28 (6.89, 9.66)	4.19 (3.23, 5.15)	20.32 (17.97, 22.67)
Southwest	7.70 (6.89, 8.51)	8.60 (7.77, 9.42)	5.59 (4.56, 6.61)	21.89 (20.23, 23.54)
Northwest	5.19 (4.43, 5.96)	8.09 (6.73, 9.44)	3.07 (2.28, 3.86)	16.34 (14.44, 18.24)

CI: confidence interval; TLE: Total life expectancy; NSLE: Non-sarcopenic life expectancy; PSLE: Possible sarcopenic life expectancy; SLE: Sarcopenic life expectancy.

Table S7. Total and sarcopenia-state life expectancy by sarcopenia state using *Simpson* method

Sarcopenia state	Total and sarcopenia-state life expectancy, years [mean (95%CI)]			
	NSLE	PSLE	SLE	TLE
**No sarcopenia**	12.03 (11.00, 13.07)	8.46 (6.90, 10.02)	1.54 (0.64, 2.43)	22.03 (19.53, 24.54)
**Possible sarcopenia**	6.40 (5.63, 7.17)	10.67 (9.80, 11.54)	3.79 (2.85, 4.73)	20.86 (19.44, 22.27)
**Sarcopenia**	2.70 (1.91, 3.48)	7.91 (6.39, 9.44)	8.06 (6.74, 9.38)	18.67 (16.30, 21.03)
**Average**	8.03 (7.59, 8.46)	8.70 (8.20, 9.20)	4.11 (3.70, 4.52)	20.84 (20.18, 21.49)

CI: confidence interval; TLE: Total life expectancy; NSLE: Non-sarcopenic life expectancy; PSLE: Possible sarcopenic life expectancy; SLE: Sarcopenic life expectancy.

Table S8. Total and sarcopenia-specific life expectancy for older Chinese at age 60 by demographic and lifestyle subgroups using *Simpson* method

Variables	Total and sarcopenia-specific life expectancy, years [mean (95%CI)]			
	NSLE	PSLE	SLE	TLE
**Sex**				
Man	8.35 (7.82, 8.89)	7.85 (7.21, 8.49)	3.05 (2.70, 3.39)	19.25 (18.39, 20.10)
Woman	7.68 (7.12, 8.23)	9.60 (9.02, 10.18)	5.50 (4.82, 6.19)	22.78 (21.75, 23.82)
**Education**				
Illiterate	7.03 (6.50, 7.57)	9.51 (8.98, 10.03)	4.76 (4.18, 5.33)	21.30 (20.5, 22.09)
Nonformal education	6.87 (6.18, 7.57)	8.31 (7.28, 9.34)	4.29 (3.47, 5.10)	19.47 (17.96, 20.97)
Elementary school	9.01 (8.07, 9.95)	8.20 (7.22, 9.17)	3.41 (2.47, 4.36)	20.62 (19.06, 22.18)
Middle school or above	10.00 (9.15, 10.85)	8.86 (7.58, 10.14)	3.21 (2.14, 4.29)	22.07 (19.91, 24.23)
**Marital status**				
Married	8.38 (7.88, 8.87)	8.65 (8.07, 9.23)	3.96 (3.55, 4.36)	20.98 (20.11, 21.85)
Unmarried	6.82 (6.11, 7.53)	8.93 (8.08, 9.78)	4.47 (3.78, 5.15)	20.21 (19.04, 21.39)
**Smoking**				
No	8.33 (7.85, 8.80)	9.68 (9.03, 10.33)	5.02 (4.43, 5.61)	23.03 (21.94, 24.11)
Former	8.06 (6.98, 9.13)	6.69 (5.63, 7.75)	1.89 (1.31, 2.48)	16.64 (15.21, 18.07)
Current	7.58 (6.82, 8.33)	8.32 (7.57, 9.06)	4.02 (3.34, 4.71)	19.92 (18.69, 21.15)
**Drinking**				
No	7.79 (7.32, 8.26)	8.97 (8.53, 9.42)	4.30 (3.86, 4.74)	21.06 (20.32, 21.81)
Yes	8.59 (7.81, 9.38)	7.97 (6.98, 8.95)	3.75 (3.07, 4.44)	20.31 (18.85, 21.77)
***Hukou* type**				
Agriculture	7.33 (6.92, 7.74)	8.85 (8.44, 9.25)	4.52 (4.09, 4.95)	20.69 (19.99, 21.39)
Non-agriculture	10.58 (9.58, 11.58)	7.93 (6.72, 9.13)	2.57 (1.96, 3.17)	21.07 (19.46, 22.69)
**Living residence**				
Rural	7.40 (6.95, 7.85)	8.79 (8.35, 9.23)	4.83 (4.32, 5.33)	21.02 (20.23, 21.81)
Urban	9.30 (8.56, 10.03)	8.37 (7.47, 9.28)	2.83 (2.39, 3.27)	20.50 (19.31, 21.69)
**Region**				
Northeast	6.78 (5.90, 7.65)	8.85 (7.52, 10.17)	3.82 (2.36, 5.28)	19.45 (17.58, 21.32)
East	9.03 (8.20, 9.86)	8.97 (8.19, 9.75)	3.79 (3.08, 4.51)	21.8 (20.56, 23.03)
North	8.93 (7.45, 10.41)	8.10 (6.85, 9.34)	3.00 (1.97, 4.03)	20.02 (17.97, 22.07)
Centre	8.29 (7.48, 9.10)	8.59 (7.41, 9.77)	3.91 (2.93, 4.89)	20.79 (19.29, 22.29)
South	7.85 (6.57, 9.12)	8.27 (6.88, 9.66)	4.19 (3.23, 5.15)	20.31 (17.95, 22.66)
Southwest	7.70 (6.89, 8.51)	8.59 (7.77, 9.42)	5.58 (4.56, 6.61)	21.88 (20.22, 23.53)
Northwest	5.18 (4.42, 5.95)	8.08 (6.73, 9.44)	3.07 (2.28, 3.85)	16.33 (14.43, 18.23)

CI: confidence interval; TLE: Total life expectancy; NSLE: Non-sarcopenic life expectancy; PSLE: Possible sarcopenic life expectancy; SLE: Sarcopenic life expectancy.
